# Cross-Sectional but Not Prospective Association of Accelerometry-Derived Physical Activity With Quality of Life in Children and Adolescents

**DOI:** 10.3389/ijph.2024.1606737

**Published:** 2024-02-19

**Authors:** Ranin Darkhawaja, Johanna Hänggi, Emmanuel Schaffner, Marek Kwiatkowski, Abdulsalam Alkaiyat, Alain Dössegger, Bengt Kayser, L. Suzanne Suggs, Bettina Bringolf-Isler, Nicole Probst-Hensch

**Affiliations:** ^1^ Department of Epidemiology and Public Health, Swiss Tropical and Public Health Institute, Allschwil, Switzerland; ^2^ Department of Public Health, University of Basel, Basel, Switzerland; ^3^ Public Health Department, Faculty of Medicine and Health Sciences, An-Najah National University, Nablus, Palestine; ^4^ Swiss Federal Institute of Sport Magglingen, Magglingen, Bern, Switzerland; ^5^ Institute of Sport Sciences, University of Lausanne, Lausanne, Vaud, Switzerland; ^6^ Institute of Communication and Public Policy, Faculty of Communication, Culture, and Society, Università della Svizzera italiana, Lugano, Ticino, Switzerland

**Keywords:** moderate-to-vigorous physical activity, quality of life, longitudinal data, linear mixed effects model, linear regression model

## Abstract

**Objectives:** This study aims to quantify the cross-sectional and prospective associations between quality of life (QoL) and moderate-to-vigorous physical activity (MVPA).

**Methods:** This study was based on the Swiss children’s Objectively measured PHYsical Activity cohort. The primary endpoint is the overall QoL score and its six dimensions. The main predictor is the average time spent in MVPA per day. Linear mixed effects and linear regression models respectively were used to investigate the cross-sectional and prospective associations between MVPA and QoL.

**Results:** There were 352 participants in the study with complete data from baseline (2013–2015) and follow-up (2019). MVPA was positively associated with overall QoL and physical wellbeing (*p* = 0.023 and 0.002 respectively). The between-subject MVPA was positively associated with the overall QoL, physical wellbeing, and social wellbeing (*p* = 0.030, 0.017, and 0.028 respectively). Within-subject MVPA was positively associated with physical wellbeing and functioning at school (*p* = 0.039 and 0.013 respectively). Baseline MVPA was not associated with QoL 5 years later.

**Conclusion:** Future longitudinal studies should employ shorter follow-up times and repeat measurements to assess the PA and QoL association.

## Introduction

In Switzerland, children and adolescents under 20 years account for approximately 20% of the population [1]. Children are a vulnerable group, thus the protection of their rights to adequate wellbeing is deemed to be important [2]. Understanding children’s [3] and adolescents’ [4] physical and mental health and their determinants is fundamental to their healthy upbringing. Investment into promoting their health and wellbeing can contribute to the achievement of several public health agendas [5].

Assessment of children’s and adolescents’ health should resonate with the comprehensive definition of health as “a state of complete physical, mental, and social wellbeing and not merely the absence of disease or infirmity” [6]. Quality of life (QoL), which is defined as “the individual’s physical health, psychological state, level of independence, social relationships, personal beliefs and his/her relationships to salient features of the environment” [7], has been suggested to be a critical indicator of health [8]. QoL is ideally measured on the basis of subjectively reported broad indicators not restricted to, medical wellbeing indicators. It is important to assess its distribution in general population samples and not only in subgroups with a specific disease [2]. The KINDL^®^ questionnaire has been proven to be a reliable and valid instrument for assessing QoL in different subdomains among children and adolescents [9–12]. It assesses the overall QoL and its six specific dimensions: physical wellbeing, emotional wellbeing, self-esteem, family connection, social wellbeing, and functioning at school [13].

Moderate-to-vigorous physical activity (MVPA) is a strong predictor for different health aspects in children and adolescents [14]. Accordingly, the World Health Organization (WHO) recommends that children and adolescents aged 5–17 years maintain an average of 1 h per day in MVPA [15]. Yet, before the COVID-19 pandemic, it was estimated that 81% of 11–17-year-old students globally were not sufficiently active [16]. Similar trends were reported among children [17]. This is of particular concern as both PA [18] and, to a much smaller extent, QoL [19] decrease with the aging of children.

A recent review summarized the evidence on the PA and QoL/wellbeing association in the general population and across the life course [20]. Among youth aged 5–18, a higher level of physical activity (PA) and less sedentary time was associated with higher QoL and wellbeing perception, confirming results from an earlier review [21]. The variety of instruments for assessing QoL and the differences in PA type considered complicate firm conclusions based on the evidence available. Most youth studies conducted to date were cross-sectional or the follow-up period in cohort and intervention studies was limited. No population-based cohort studies that report on the PA-QoL association in the young measured PA with the help of accelerometry [20, 21].

The first meta-analysis of the effects of PA interventions on health-related quality of life (HRQoL) in healthy children and adolescents found PA to improve HRQoL overall and in several domains [22]. The authors concluded that considering a) the limited number of studies (*N* = 17) and b) the large heterogeneity of the interventions there is insufficient evidence on the type and duration of PA intervention needed to benefit HRQoL in children and adolescents.

The current study benefits from accelerometry-derived PA and QoL assessment with the validated KINDL^®^ questionnaire measured twice over a follow-up period of 5 years in the population-based Swiss children’s Objectively measured PHYsical Activity (SOPHYA) cohort study. The study’s overall objective was to investigate associations between accelerometry-derived MVPA, given the specific WHO recommendations for this PA indicator, and QoL, with a focus on the longitudinal aspects. The following specific main research questions were addressed: 1) Is MVPA associated cross-sectionally with the overall QoL and the specific dimensions of QoL? 2) To what extent are these associations driven by the between-subject or within-subject variability in MVPA? 3) Does MVPA measured at baseline predict the overall QoL or/and the specific dimensions of QoL 5 years later independent of baseline participants’ characteristics?

## Methods

### Study Design and Population

**FIGURE 1:**
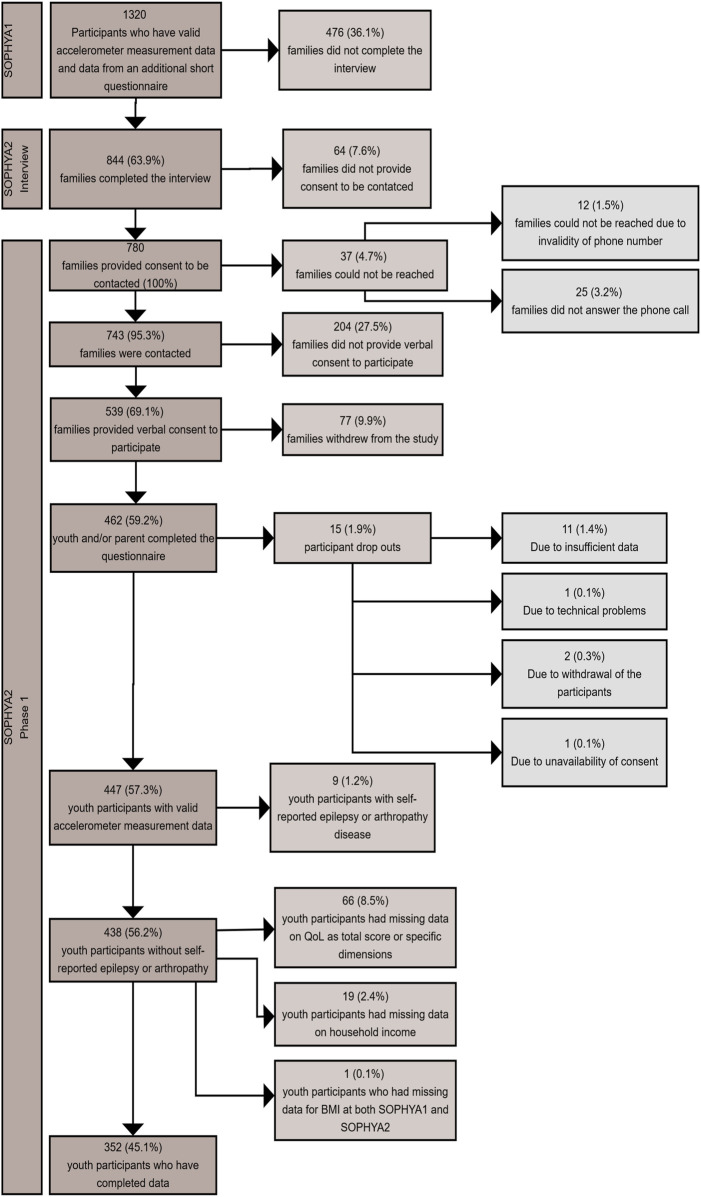
Flow diagram of the study population (Swiss children’s Objectively measured PHYsical Activity cohort study, Switzerland, 2013–2019).

The present study was conducted among children and adolescents participating in the baseline assessment of the SOPHYA cohort (SOPHYA1) between December 2013 and June 2015 [23]. All youth who are registered as residents in Switzerland and born between 1998 and 2007 were eligible. The Federal Statistical Office drew random samples from this sampling frame stratified by sex, year of birth, and language (German; French; Italian). The recruitment and the participation rate in SOPHYA1 were described before [23]. In short, the participation rate among 2,032 families who could be contacted for an accelerometer measurement and answered the SOPHYA1 baseline interview, was 64.4%. Valid accelerometer measurements accompanied by a self-administrated questionnaire during the measurement week were obtained from 1,320 youth aged 6–16 years (Figure 1).

For the assessment of the predictive association of MVPA at baseline, QoL data obtained at the follow-up assessment in 2019 (SOPHYA2 accelerometry) was considered as outcome. SOPHYA2 was based on the 1,320 SOPHYA1 baseline accelerometry participants who provided proxy-reported (parents) information on socio-demographic characteristics, weight, height, and QoL. Of these participants, 844 could be re-contacted by phone in 2019 and 780 provided consent for a follow-up accelerometer measurement. Among them, 447 participants finally had valid accelerometer measurements and self-reported socio-demographic characteristics, weight, height, and QoL (Figure 1).

In SOPHYA1, parents gave written informed consent (IC) for their children’s participation. Adolescents aged 12 years or older filled in an additional IC form. In SOPHYA2, for participants younger than 14 years written IC was provided by a parent as proxy; for participants aged between 14 and 18 years, both parental and an own written IC was provided; for youth above 18 years only own written IC was given.

### Data Collection

Since participants were spread across Switzerland, contact with them was exclusively remote by phone and by mail. The regional SOPHYA-study partners (German-speaking region: Swiss Tropical and Public Health Institute in Basel; French-speaking regions: University of Lausanne; Italian-speaking regions: Università della Svizzera Italiana) coordinated participant assessment.

#### Telephone Interview

At baseline and follow-up as a first SOPHYA assessment, computer-assisted telephone interviews in the respective language region (German; French; Italian) were conducted by a professional field research institute (LINK institute, Lucerne, Switzerland) either directly with children 11 years or older or with one parent as proxy for children aged 10 years or younger [24]. Interview data collected included sociodemographic characteristics (sex, nationality, and household income).

#### Accelerometer Measurement

In SOPHYA1 and SOPHYA2, families were given oral instructions on accelerometer use through a phone call. An accelerometer along with written instructions was subsequently mailed to the participants, together with a pre-paid postage box to return the devices to the investigators after completion of the measurements. The participants mainly wore either Actigraph accelerometer model GT3X (exclusively used in SOPHYA2) and a few wore GT1M in SOPHYA1 (out of 1,320 participants; 49 wore GT1M and 1,271 wore GT3X), (ActiGraph, Pensacola, Florida, United States), both producing comparable output [25]. Only the vertical axis was used when the accelerometer data was analyzed regardless of the type of the accelerometer being used. The accelerometers were tied to the right hip with an elastic band for seven consecutive days. The device was not worn during water activities or sleep. The device was set without filtering and in 15-s epoch mode in order to detect shorter bursts of MVPA, which are typical for children [26]. ActiLife 6.2 software (ActiGraph, Pensacola, Florida, United States) was used to initialize the device before wearing and for downloading and processing the data. Any period of 60 or more minutes of consecutive zero counts was considered non-wearing time. A day was considered valid if it contained at least 10 or 8 h of wear time on weekdays or weekend days, respectively. Valid accelerometry consisted of at least three valid weekdays and one weekend day. To define children’s time spent in MVPA, the age-dependent cut-offs of Freedson [27] with a threshold of four metabolic equivalents were used [28].

#### Paper-Based Survey

In SOPHYA1 and SOPHYA2, families who had agreed to participate in the accelerometry sub-study received an additional paper-based survey to answer questions on the child’s age when the accelerometer measurements took place, sports behavior of the children during the measured week, their weight, their height, and any diagnosis of chronic disease. Additionally, the survey included the validated KINDL^®^ questionnaire [29] for assessing children’s QoL. The questionnaire was administered in the three language areas in Switzerland using the official translation of the questionnaire (Romansh-speaking people filled in the German questionnaire). Validated questionnaire versions tailored to different age groups are available for self-assessment and as a parent-proxy tool [13]. In SOPHYA1, the questionnaire was exclusively answered by a parent. At follow-up in SOPHYA2, questionnaires were answered additionally by the participants themselves given their older age.

### Measures

#### Primary Endpoint: QoL

The validated KINDL^®^ QoL questionnaire consists of 24 items, each answered on a five-point ordinal Likert scale ranging from “never” = (5) to “always” = (1). Each item belongs to one of the six QoL dimensions (four items per dimension): physical wellbeing, emotional wellbeing, self-esteem, family connection, social wellbeing, and functioning at school. The QoL dimensions are scored separately as the sum of the scores of 4 items, ranging from 4 to 20. The domain-specific scores are subsequently transformed to a scale from 0 to 100. The overall QoL score is calculated based on the mean value of all answered items. Higher scores represent a higher QoL. If missing values occurred and affected less than 70% of the answers contributing to a dimension or the total score, the algorithm proposed by the authors of the KINDL^®^ questionnaire was used to replace these missing data [30]. The exclusion of participants affected only 2.0% in SOPHYA1 and 2.3% in SOPHYA2 [31].

#### Main Predictor: Mean of MVPA in Hours per Day

Accelerometer data averaged over a week was used to derive the participant’s mean hours per day spent exercising in MVPA. To account for different numbers of valid weekdays and weekend days and to reflect that potential differences in MVPA between weekdays and weekends were considered when estimating average MVPA spent per day in the light of missing weekdays or weekend days, respectively, mean MVPA over the week was calculated as follows: (Average MVPA spent per day for weekdays * 5) + (Average MVPA spent per day for weekend days * 2)/7. MVPA was defined by age-related cut points with a threshold for moderate activity of 4 MET [27, 28].

#### Confounders

**- Sociodemographic characteristics**

A telephone interview was used to collect information on the participant’s sex (*male, female*), nationality (*Swiss, foreign nationality, Swiss dual citizen*), language region (*German, French, Italian*), and household income (less or equal to 6,000 CHF, 6,001 to 9,000 CHF, 9,001 and more CHF, no information provided). Age (years) at the time of measurement was assessed by subtracting the date of birth from the date of measurement recorded by the paper-based survey during the measurement week.

**- Body Mass Index (BMI)**

Based on the paper-based survey, the participants self-reported their height and weight. BMI was calculated based on the following equation:

$$\text{Weight}\left(\text{kg}\right)/{\text{height}}^{2}\left(m^{2}\right)$$

For sensitivity analysis, BMI-for-age percentiles were calculated.

**- Health status**

In their responses to the paper-based survey, participants self-reported the following chronic disease diagnoses: asthma, hay fever, allergy, atopic dermatitis, diabetes mellitus, chronic enteritis, hypertension, epilepsy, arthropathy, and attention deficit hyperactivity disorder. They also self-reported the presence of any other chronic disease not specifically included in the above-mentioned list. Self-reported diagnosis of at least one chronic disease was defined as having had at least one chronic disease.

**- Use of the accelerometer**

The season of the wear time was assigned based on the month of accelerometer measurement (Spring: March-May; Summer: June-August; Autumn: September-November; Winter: December-February).

### Statistical Analysis

Participants included in the analysis were required to have complete data from both time points for the overall QoL and its specific dimensions (physical wellbeing, emotional wellbeing, self-esteem, family connection, social wellbeing, and functioning at school), MVPA, BMI, and for the selected covariates (sex, age, household income, language region, nationality, self-reported diagnosis of chronic disease, and season of measurement). If the BMI of the participant was missing at one time point only, it was imputed by regressing it on the BMI value available from the other time point while adjusting for age, sex, and time interval between the two time points (See Result Section). Participants self-reporting epilepsy or arthropathy were excluded from the study, because of their strong potential influence on both PA and QoL [32, 33].

The flow diagram of the study population is presented in Figure 1.

Potential selection bias was assessed by comparing baseline characteristics of all SOPHYA1 participants with those retained for the current study using chi-squared tests and Student’s t-tests (Supplementary Table S1).

Descriptive statistics (*n*, %, mean, SD) of the study population, QoL and its specific dimensions, and of MVPA, BMI, and covariates were calculated for the total study sample, and for SOPHYA1 and SOPHYA2 separately (Table 1).

**TABLE 1:** Characteristics of study participants at baseline (SOPHYA1; 2013–2015) and follow-up (SOPHYA2; 2019) [Swiss children’s Objectively measured PHYsical Activity cohort study (SOPHYA), Switzerland, 2013–2019].

N = 352		
	SOPHYA 1	SOPHYA 2
	Mean (SD)/N (%)	Mean (SD)/N (%)
Socio-demographic		
Age	10.3 (2.4)	15.1 (2.6)
Sex		
- Male	166 (47.2%)	
- Female	186 (52.8%)	
Household income^a^		
- ≤ 6,000 CHF	66 (18.8%)	
−6,001 to 9,000 CHF	115 (32.7%)	
−9,000 and more CHF	158 (44.9%)	
- Not willing to provide information^b^	13 (3.7%)	
Language region^a^		
- German	245 (69.6%)	
- French	74 (21.0%)	
- Italian	33 (9.4%)	
Nationality^a^		
- Swiss	245 (69.6%)	
- Foreign nationality	33 (9.4%)	
- Swiss dual citizen (Swiss and foreign nationality)	74 (21.0%)	
Health status		
BMI kg/m^2^	16.8 (2.4)	19.8 (3.1)
Self-reported diagnosis with at least one chronic disease		
- Did not have any of the chronic diseases	254 (72.2%)	232 (65.9%)
- Had at least one chronic disease	98 (27.8%)	120 (34.1%)
Physical activity		
Season of measurement		
- Spring	85 (24.1%)	104 (29.5%)
- Summer	54 (15.3%)	73 (20.7%)
- Autumn	92 (26.1%)	127 (36.1%)
- Winter	121 (34.4%)	48 (13.6%)
Weartime hour/day	13.2 (0.9)	13.9 (1.1)
MVPA Mean hour/day	1.4 (0.6)	0.8 (0.4)
Quality of life^c^		
- Overall QoL	82.3 (7.7)	74.2 (9.9)
- Physical wellbeing	84.9 (12.6)	74.2 (14.9)
- Emotional wellbeing	87 (10.6)	79.7 (13.0)
- Self-esteem	77.3 (12.4)	60.9 (17.3)
- Family connection	82.5 (12.0)	86.3 (14.1)
- Social wellbeing	79.2 (11.2)	74.3 (14.0)
- Functioning at school	82.9 (14.2)	69.6 (17.6)

^a^ The information for both time points stems from baseline assessment.

^b^ Participant answered the question, but chose to abstain from declaring the range of the household income.

^c^ Obtained from KINDL^®^ questionnaire.

The selection of the covariates as potential confounders was done *a priori*. We adjusted all models for age, sex, household income, language region, nationality, self-reported diagnosis with a chronic disease, and season of measurement.

We assessed whether MPVA was cross-sectionally or longitudinally associated with QoL and its specific dimensions by regressing QoL on MVPA while adjusting for the above-mentioned covariates. The following models were sequentially applied.

First, in Model 1 (Table 2) we assessed whether MVPA was associated cross-sectionally with QoL. We considered data from both time points (SOPHYA1 and SOPHYA2). We used a linear mixed model that included a subject-specific random intercept.

**TABLE 2:** Repeated adjusted^a^ cross-sectional association of mean moderate-tovigorous physical activity with quality of life (Swiss children’s Objectively measured PHYsical Activity cohort study, Switzerland, 2013–2019).

Model 1^b^			
MVPA			
Primary endpoint^c^	Coefficient^d^	95% CI	*p*-value
Overall QoL	2.0	(0.3 to 3.7)	0.023
Physical wellbeing	4.2	(1.6 to 6.8)	0.002
Emotional wellbeing	1.7	(−0.6 to 4.0)	0.151
Self-esteem	1.4	(−1.4 to 4.4)	0.335
Family connection	−0.6	(−3.0 to 1.9)	0.660
Social well-being	2.4	(0.002 to 4.9)	0.053
Functioning at school	2.6	(−0.4 to 5.6)	0.086

^a^ Adjusted for age, sex, household income, language region, nationality, diagnosis with a chronic disease, and season of measurement.

^b^ Moderate-to-vigorous physical activity at the respective time point.

^c^ Obtained from KINDL^®^ questionnaire.

^d^ Coefficient reflects the change in score associated with an average 1-hour increase in moderate-to-vigorous physical activity during the accelerometry measurement week.

Second, in Model 2 (Table 3) we assessed the relationship of between- and within-subject variation in MVPA with the variation in QoL. The between-subject MVPA value for each participant was defined as their individual mean, which is the average of their MVPA over the two time points. The participant’s within-subject MVPA value at each time point was defined as the deviation of MVPA as measured at that point from the participant’s individual mean. We included both the between- and within-subject MVPA instead of MVPA in linear mixed models mirroring model 1. This modeling approach is described in more detail in [34].

**TABLE 3:** Repeated adjusted^a^ cross-sectional association of between-and within-subject moderate-to-vigorous physical activity values with quality of life (Swiss children’s Objectively measured PHYsical Activity cohort study, Switzerland, 2013–2019).

Model 2^b^						
	MVPA—Between subjects			MVPA—Within subjects		
Primary endpoint^c^	Coefficient^d^	95% CI	*p*-value	Coefficient^e^	95% CI	*p*-value
Overall QoL	2.6	(0.3 to 4.9)	0.030	1.3	(−1.1 to 3.7)	0.302
Physical wellbeing	4.3	(0.8 to 7.7)	0.017	4.2	(0.2 to 8.2)	0.039
Emotional wellbeing	2.7	(−0.3 to 5.7)	0.085	0.4	(−3.0 to 3.8)	0.804
Self-esteem	3.5	(−0.4 to 7.4)	0.084	−1.0	(−5.3 to 3.3)	0.649
Family connection	1.2	(−2.3 to 4.7)	0.510	−2.3	(−5.8 to 1.2)	0.205
Social wellbeing	3.8	(0.5 to 7.2)	0.028	0.9	(−2.7 to 4.5)	0.623
Functioning at school	0.2	(−3.8 to 4.1)	0.938	5.6	(1.2 to 10.1)	0.013

^a^ Adjusted for age, sex, household income, language region, nationality, diagnosis with a chronic disease, and season of measurement.

^b^ Moderate-to-vigorous physical activity included in the model as participant’s mean moderate-to-vigorous physical activity over both time points (between-subject variation) and as difference from that mean at either time point (within-subject variation).

^c^ Obtained from KINDL^®^ questionnaire.

^d^ Coefficient reflects the change in score associated with an average 1-hour increase in between-subject moderate-to-vigorous physical activity during the accelerometry measurement week.

^e^ Coefficient reflects the change in score associated with an average 1-hour increase in within-subject moderate-to-vigorous physical activity during the accelerometry measurement week.

Third, in Model 3 (Table 4) we assessed whether MVPA measured at baseline predicted QoL after 5 years of follow-up. QoL scores at follow-up were regressed on MVPA at baseline in the context of a linear regression model adjusting for covariates as described above and in addition to the respective QoL scores at baseline.

**TABLE 4:** Prospective adjusted^a^ association of mean of moderate-to-vigorous physical activity at baseline with quality of life (overall and specific dimension scores) at follow-up (Swiss children’s Objectively measured PHYsical Activity cohort study, Switzerland, 2013–2019).

Model 3			
Main predictor			
MVPA			
Primary endpoint^b^	Coefficient^c^	95% CI	*p*-value
Overall QoL	−0.9	(−3.5 to 1.6)	0.479
Physical wellbeing	−0.7	(−4.6 to 3.2)	0.730
Emotional wellbeing	0.1	(−3.2 to 3.5)	0.946
Self-esteem	−0.1	(−4.6 to 4.5)	0.980
Family connection	−1.0	(−4.6 to 2.7)	0.598
Social wellbeing	−0.1	(−3.8 to 3.6)	0.971
School functioning	−2.9	(−7.5 to 1.8)	0.226

^a^ Adjusted for age, gender, household income, language region, nationality, diagnosed with a chronic disease, season of measurement, and respective quality of life score at baseline.

^b^ Obtained from KINDL^®^ questionnaire.

^c^ Coefficient reflects the change in score associated with an average 1-hour increase in within-subject moderate-to-vigorous physical activity during the accelerometry measurement week.

Fourth, a secondary analysis (Supplementary Tables S2–S4), models 1–3 described above were re-fitted with an additional adjustment for BMI to assess the independence of the study’s associations from the participants’ BMI.

The association size in Models 1–3 represented by the regression coefficient reflects the change in QoL (sub)-score for an increase in MVPA by an average of 1 h/day during the measurement week.

For sensitivity analyses, models 1 to 3 were repeated 1) using QoL at follow-up derived from parental-proxy questionnaire instead of youth self-report at follow-up, and 2) using BMI_for_age percentiles instead of BMI for additional adjustment. As the sample sizes for parental-proxy-derived QoL (*N* = 302) and percentiles (*N* = 341) were only available from a smaller follow-up sample, the sensitivity analyses were conducted on these smaller samples.

All analyses were performed in R version 4.1.3.

## Results

The final sample consisted of 352 children and adolescents (Figure 1). Of the 447 SOPHYA1 and SOPHYA2 accelerometry participants, 66 (8.5%) participants were not included in the final analysis because they did not have valid QoL scores (overall or specific dimension). Additionally, 19 (2.4%) participants were dropped because they did not have valid data on household income. BMI at baseline or follow-up was imputed for 28 participants (11 participants did not have valid BMI in SOPHYA1 and 17 participants did not have valid BMI in SOPHYA2). One participant (1; 0.1%) was excluded for not having valid BMI information for either time point. Participants who self-reported a diagnosis of epilepsy 2 (0.3%) or arthropathy 7 (0.9%) in either SOPHYA1 or SOPHYA2 were also excluded from this study analysis.

Potential selection bias was assessed by comparing the baseline characteristics of all SOPHYA1 accelerometry participants with those retained for the current study using chi-squared tests and Student’s t-test. A comparison of baseline characteristics between participants not included versus those who are included in this current analysis is presented in Supplementary Table S1. Participants included in this analysis exhibited a lower BMI at baseline (mean [SD]: 16.8 [2.4] vs. 17.7 [2.9], *p* < 0.0001), higher MVPA levels (mean [SD]: 1.4 [0.6] vs. 1.2 [0.6], *p* < 0.0001) and better overall QoL (mean [SD]: 82.3 [7.7] vs. 80.2 [9], *p* < 0.0001), including better physical wellbeing (mean [SD]: 84.9 [12.6] vs. 83.2 [14.6], *p* = 0.041), self-esteem (mean [SD]: 77.3 [12.4] vs. 74.6 [14.9], *p* = 0.001), and functioning at school (mean [SD]: 82.9 [14.2] vs. 78.4 [15.5], *p* < 0.0001).

Table 1 describes the study sample. The final sample consisted of 52.8% females and 47.2% males. The average ages of the participants in SOPHYA1 and SOPHYA2 were (mean [SD]: 10.3 [2.4] years) and (mean [SD]: 15.1 [2.6] years), respectively. Mean MVPA decreased from (mean [SD]: 1.4 [0.6] hr/day) in SOPHYA1 to (mean [SD]: 0.8 [0.4] hr/day) in SOPHYA2. In regards to the QoL, the average score of the overall QoL decreased from (mean [SD]: 82.3 [7.7]) in SOPHYA1 to (mean [SD]: 74.2 [9.9]) in SOPHYA2. Of the specific QoL dimensions, self-esteem exhibited the lowest score at both time points and decreased from (mean [SD]: 77.3 [12.4]) to (mean [SD]: 60.9 [17.3]) over 5 years of follow-up. Males and females were comparable on all characteristics except for the mean of MVPA and the mean of QoL score. Males had significantly higher MVPA (mean [SD]: 1.6 [0.7] vs. 1.3 [0.5], *p* < 0.0001) in SOPHYA1 and (mean [SD]: 0.9 [0.4] vs. 0.7 [0.3]), *p* < 0.0001 in SOPHYA2. Overall QoL was lower in females (mean [SD]: 73.1 [10.1] vs. 75.4 [9.5], *p* = 0.026) in SOPHYA2 only.

### Cross-Sectional Associations

The results of the cross-sectional analysis of data obtained from the same children at baseline and follow-up on the adjusted association between mean MVPA and QoL are shown in Table 2. Mean MVPA was positively associated with the overall QoL and with physical wellbeing (*p* = 0.023 and 0.002 respectively). A 1-hour increase in MVPA per day was associated with a 2.0 (95%CI: 0.3, 3.7) points and 4.2 (95%CI: 1.6, 6.8) points increase in the overall QoL and physical wellbeing score, respectively. Positive but statistically non-significant associations were also observed for social wellbeing and functioning at school (*p* = 0.053 and 0.086 respectively) with an effect size of 2.4 (95%CI: 0.002, 4.9) points and 2.6 (95%CI: −0.4, 5.6) points, respectively per each 1-hour increase in MVPA. No associations were observed with other dimensions of QoL (*p* = 0.151).

The between-subject MVPA value, reflecting the cross-sectional association, was positively associated with the overall QoL, physical wellbeing, and social wellbeing (*p* = 0.030, 0.017, and 0.028 respectively). For every 1-h increase in the between-subject MVPA value, there was a 2.6 (95%CI: 0.3, 4.9) points, 4.3 (95%CI: 0.8, 7.7) points, and 3.8 (95%CI: 0.5, 7.2) points increase in the overall QoL, the physical wellbeing, and the social wellbeing, respectively. The within-subject MVPA value, reflecting the longitudinal association, was positively associated with the physical wellbeing and functioning at school (*p* = 0.039 and 0.013 respectively), with effect sizes of 4.2 (95%CI: 0.2, 8.2) points and 5.6 (95%CI: 1.2, 10.1) points increase in physical wellbeing and functioning at school scores, respectively, for every 1-h increase in the within-subject MVPA. See Table 3.

The results presented in Table 2 and Table 3 did not materially change upon additional adjustment for BMI (Supplementary Tables S2, S3).

### Prospective Associations

We did not find evidence of an association of MVPA at baseline with QoL 5 years later, after adjusting for the baseline characteristics, including baseline QoL (Table 4). Again, results were not materially altered by an additional adjustment for BMI (Supplementary Table S4).

### Sensitivity Analyses

Replacing QoL follow-up information provided by children and adolescents themselves with QoL information provided by parental proxy did not materially alter the results reported. Replacing additional adjustment for BMI with adjustment for BMI-for-age percentiles did not change the conclusion.

## Discussion

This study demonstrates a cross-sectional, positive association of device-based MVPA with both overall QoL and physical wellbeing of Swiss children and adolescents. Partitioning of the variation in MVPA into between- and within-subject variation to differentiate between cross-sectional and longitudinal associations revealed that the latter is associated with the functioning at school dimension of QoL. As another approach to assessing longitudinal associations, baseline MVPA did not predict a better QoL in any specific dimension 5 years later.

The cross-sectional association between QoL and PA is in agreement with many findings of previous cross-sectional studies in children and adolescents, which were based on device-based PA and self-reported PA or sports activities, and applied different QoL instruments [20, 35]. Differences in observed associations with QoL-specific dimensions across studies may in part be due to differences in PA assessment. This is evidenced by a cross-sectional study conducted among adolescents in Germany to compare the QoL association with self-reported PA versus device-based PA assessment. While self-reported PA was associated with almost all specific dimensions of QoL, device-based PA was mostly associated with physical wellbeing [35]. Furthermore, sports activity and PA more generally seem to have different influences on QoL [36, 37]. Observed associations in cross-sectional studies do not inform the direction of associations and are primarily based on between-subject effects. The results of PA intervention trials support positive short-term effects on children’s QoL [22].

Additionally, we attempted to characterize the relationship between MVPA and QoL from two distinct longitudinal perspectives. First, employing a conventional epidemiological analysis, we did not find evidence of an effect of baseline PA on QoL at the follow-up 5 years later. While such an effect is plausible, our study may have lacked the statistical power to detect it. This is especially likely given the long follow-up consisting of our young participants’ formative years which would have attenuated the effect size. Longitudinal evidence of the longer-term benefit of PA on QoL in children and adolescents is still rare and absent to our knowledge for accelerometry-derived MVPA. Several prospective and interventional studies reported different types of PA to be a predictor of QoL among children and adolescents [38, 39]. However, PA was mostly self-reported and related to engagement in sports activities or school interventions. A few longitudinal studies with data from more than two time points specifically investigated the bidirectional association between PA and QoL in children and adolescents using cross-lagged panel models. Jensen et al. (2014) found higher baseline QoL to predict higher PA 1 year later [40]. Wunsch et al. (2021) found pre-pandemic HRQoL to predict higher PA during the pandemic, but only in children and not in adolescents [41]. Consistent with our results, neither of the two studies above found PA to predict HRQoL. In contrast, a positive predictive effect of PA on HRQoL 1 year later in the absence of a predictive effect of HRQoL on PA was reported for Finnish adolescents aged 11–15 years [42]. A bidirectional predictive association was reported in French adolescents based on self-reported PA obtained in adolescents assessed over three 1-year follow-ups [43].

The second perspective related deviations of participants’ MVPA at either time point from their five-year mean to their QoL scores. Here, notably, we observed an association with the functioning at school domain. It is plausible that an increased PA enables a child to feel more comfortable at school, either directly or through the multitude of its physical and mental health benefits. Another possibility is that a decreased PA is a marker of a lowered school-related QoL. The associations may also be artifacts arising from complex interplays of parallel downward trends in PA and QoL over the study period, regression to the mean, and the 5-year mean of MVPA being an imprecise characterization of the child’s typical behavior.

Recently, a study on children applied a cross-lagged panel model to distinguish both between- and within-person variance to investigate prospective within-person effects between PA and HRQoL [44]. According to this study, positive deviations from an individual’s level of PA were followed by a positive deviation in the individuals’ level of HRQoL at the next measurement occasion and *vice versa*.

The strengths of this study include its population-based design covering a whole country. It is to our knowledge the first population-based youth cohort longitudinally assessing the association of accelerometry-derived MVPA with QoL over an extended period of time. QoL was assessed with the help of the widely used and validated KINDL^®^ instrument.

The study has several limitations. First, the sample size was limited as the study was embedded into a cohort with the primary aim to study the longitudinal course of PA and its determinants. Accordingly, no sample size calculations for the current objective were conducted. Second, bias due to follow-up cannot be excluded and its direction is difficult to judge given that both physical activity and QoL influenced follow-up participation. Third, two follow-up time points 5 years apart are not sufficient to clearly differentiate between predictor (MVPA) and outcome. The observed difference in within-subject variation in MVPA and QoL associations and predictive MVPA and QoL associations may reflect this fact.

### Conclusion

The results support the positive cross-sectional association between PA and QoL among children and adolescents. Future longitudinal studies employing shorter follow-up times and repeat measurements can shed light on the direction of the PA and QoL association.

## Abbreviations

BMI, Body Mass Index; HRQoL, Health Related Quality of Life; MVPA, Moderate-to-vigorous physical activity; PA, Physical activity; QoL, Quality of life; SOPHYA, Swiss children’s Objectively measured PHYsical Activity; WHO, World Health Organization.
