# Supplementary material for: Cross-Sectional but Not Prospective Association of Accelerometry-Derived Physical Activity With Quality of Life in Children and Adolescents
Source: Int J Public Health. 2024 Feb 19;69:1606737. doi: 10.3389/ijph.2024.1606737 (PMC10909831; doi:10.3389/ijph.2024.1606737)
Supplement: Supplementary file 1 [file DataSheet1.docx]

**International Journal of Public Health**

**Cross-sectional and prospective association of quality of life with accelerometer-derived physical activity: the SOPHYA cohort of Swiss children and adolescents**

**Supplement Table 1. Comparison of baseline characteristics of participants with accelerometry only participants in SOPHYA1^[[1]](#endnote-1)^ with those participants in SOPHYA1 and SOPHYA2 (SOPHYA, Switzerland, 2013 – 2019)**

|  | Children who participated only in SOPHYA1  N = 781 | Children who participated in SOPHYA1 & SOPHYA2  N = 352 |  |  |
| --- | --- | --- | --- | --- |
| Sociodemographic characteristics | | | | |
| Variable | Mean (SD) | Mean (SD) | 95% CI | P-value^[[2]](#endnote-2)^ |
| Age | | | | |
| *Children and adolescents (6 to 16 years old)* | 11.4 (2.6) | 10.3 (2.4) | (-1.4 to -0.8) | < 0.001 |
| Variable | n (%) | n (%) | x-squared | P-value^[[3]](#endnote-3)^ |
| Sex | | | | |
| *- Female* | 391 (50.1%) | 186 (52.8%) | 0.6 | 0.423 |
| *- Male* | 390 (49.9%) | 166 (47.2%) |  |  |
| Household income | | | | |
| *- ≤ 6,000 CHF* | 188 (24.1%) | 66 (18.8%) | 7.3 | 0.063 |
| *- 6,001 to 9,000 CHF* | 274 (35.1%) | 115 (32.7%) |  |  |
| *- 9,000 and more CHF* | 289 (37.0%) | 158 (44.9%) |  |  |
| *- Not willing to provide information^[[4]](#endnote-4)^* | 30 (3.8%) | 13 (3.7%) |  |  |
| Language region | | | | |
| *- German* | 547 (70.0%) | 245 (69.6%) | 1.7 | 0.431 |
| *- French* | 145 (18.6%) | 74 (21.0%) |  |  |
| *- Italian* | 89 (11.4%) | 33 (9.4%) |  |  |
| Nationality | | | | |
| *- Swiss* | 536 (68.6%) | 245 (69.6%) | 0.2 | 0.917 |
| *- Foreign nationality* | 79 (10.1%) | 33 (9.4%) |  |  |
| *- Swiss dual citizen (Swiss and foreign nationality)* | 166 (21.3%) | 74 (21.0%) |  |  |
| Health status | | | | |
| Diagnosed with chronic diseases | | | | |
| *Astham* | | | | |
| *- No* | 730 (93.5%) | 326 (92.6%) | 0.2 | 0.687 |
| *- Yes* | 51 (6.5%) | 26 (7.4%) |  |  |
| *Hay fever* | | | | |
| *- No* | 659 (84.4%) | 309 (87.8%) | 2.0 | 0.158 |
| *- Yes* | 122 (15.6%) | 43 (12.2%) |  |  |
| *Allergy (other than hay fever)* | | | | |
| *- No* | 688 (88.1%) | 314 (89.2%) | 0.2 | 0.659 |
| *- Yes* | 93 (11.9%) | 38 (10.8%) |  |  |
| *Atopic dermatitis* | | | | |
| *- No* | 722 (92.4%) | 322 (91.5%) | 0.2 | 0.659 |
| *- Yes* | 59 (7.6%) | 30 (8.5%) |  |  |
| *Diabetes mellitus* | | | | |
| *- No* | 780 (99.9%) | 352 (100.0%) | <0.01 | 1.000 |
| *- Yes* | 1 (0.1%) | 0 (0.0%) |  |  |
| *Chronic enteritis (colitis ulcerosa, morbus Crohn)* | | |  |  |
| *- No* | 779 (99.7%) | 352 (100.0%) | 0.03 | 0.853 |
| *- Yes* | 2 (0.3%) | 0 (0.0%) |  |  |
| *Hypertension* | | | | |
| *- No* | 780 (99.9%) | 351 (99.7%) | <0.01 | 1 |
| *- Yes* | 1 (0.1%) | 1 (0.3%) |  |  |
| *Attention deficit hyperactivity disorder (ADHS/ADS)* | | | | |
| *- No* | 757 (96.9%) | 346 (98.3%) | 1.3 | 0.259 |
| *- Yes* | 24 (3.1%) | 6 (1.7%) |  |  |
| *Other specified sever chronic diseases* | | | | |
| *- No* | 758 (97.1%) | 343 (97.4%) | 0.03 | 0.864 |
| *- Yes* | 23 (2.9%) | 9 (2.6%) |  |  |
| Diagnosed with at least one chronic disease | | | | |
| - *Did not have any of the chronic diseases* | 521 (66.7%) | 254 (72.2%) | 3.1 | 0.079 |
| *- Had at least one*  *chronic disease* | 260 (33.3%) | 98 (27.8%) |  |  |
| Variable | Mean (SD) | Mean (SD) | 95% CI | P-valuea |
| BMI | | | | |
| - *BMI kg/m^2^* | 17.7 (2.9) | 16.8 (2.4) | (-1.2 to -0.5) | < 0.001 |
| Physical activity | | | | |
| Variable | n (%) | n (%) | x-squared | P-value |
| Season of measurement | | | | |
| *- Spring* | 241 (30.9%) | 85 (24.1%) | 5.9 | 0.117 |
| *- Summer* | 101 (12.9%) | 54 (15.3%) |  |  |
| *- Autumn* | 180 (23.0%) | 92 (26.1%) |  |  |
| *- Winter* | 259 (33.2%) | 121 (34.4%) |  |  |
| Variable | Mean (SD) | Mean (SD) | 95% CI | P-valuea |
| Mean of MVPA | | | | |
| - *MVPA Mean hr/day* | 1.2 (0.6) | 1.4 (0.6) | (0.1 to 0.3) | < 0.001 |
| Quality of life^[[5]](#endnote-5)^ | | | | |
| - *Overall QoL* | 80.2 (9.0) | 82.3 (7.7) | (1.1 to 3.1) | < 0.001 |
| - *Physical well-being* | 83.2 (14.6) | 84.9 (12.6) | (0.1 to 3.4) | 0.041 |
| - *Emotional well-being* | 85.8 (11.5) | 87.0 (10.6) | (-0.3 to 2.5) | 0.122 |
| - *Self-esteem* | 74.6 (14.9) | 77.3 (12.4) | (1.0 to 4.4) | 0.001 |
| - *Family connection* | 81.0 (13.0) | 82.5 (12) | (-0.1 to 3.0) | 0.075 |
| - *Social well-being* | 78.2 (13.4) | 79.2 (11.2) | (-0.5 to 2.5) | 0.178 |
| - *Functioning at school* | 78.4 (15.5) | 82.9 (14.2) | (2.5 to 6.3) | < 0.001 |

**Supplement Table 2. Repeated adjusted^[[6]](#endnote-6)^ cross-sectional association of moderate- to-vigorous physical activity with quality of life, additionally adjusted for body mass index^[[7]](#endnote-7)^ (SOPHYA, Switzerland, 2013 – 2019)**

| Model 1^[[8]](#endnote-8)^ | | | | | | |
| --- | --- | --- | --- | --- | --- | --- |
|  | MVPA | | | BMI | | |
| Primary endpoint^[[9]](#endnote-9)^ | Coefficient^[[10]](#endnote-10)^ | 95% CI | P-value | Coefficient | 95% CI | P-value |
| Overall QoL | 1.9 | (0.2 to 3.6) | 0.027 | -0.2 | (-0.5 to 0.01) | 0.065 |
| Physical well-being | 4.2 | (1.6 to 6.8) | 0.002 | -0.02 | (-0.4 to 0.4) | 0.924 |
| Emotional well-being | 1.6 | (-0.6 to 3.9) | 0.161 | -0.2 | (-0.5 to 0.2) | 0.299 |
| Self-esteem | 1.3 | (-1.5 to 4.3) | 0.368 | -0.5 | (-1.0 to -0.1) | 0.028 |
| Family connection | -0.6 | (-3.1 to 1.9) | 0.616 | -0.3 | (-0.7 to 0.05) | 0.088 |
| Social well-being | 2.4 | (-0.04 to 4.9) | 0.058 | -0.3 | (-0.7 to 0.1) | 0.175 |
| Functioning at school | 2.6 | (-0.4 to 5.5) | 0.092 | -0.2 | (-0.7 to 0.3) | 0.410 |

**Supplement Table 3. Repeated adjusted^[[11]](#endnote-11)^ cross-sectional association of partitioned moderate- to-vigorous physical activity with quality of life, additionally adjusted for body mass index^[[12]](#endnote-12)^ (SOPHYA, Switzerland, 2013 – 2019)**

| Model 2^[[13]](#endnote-13)^ | | | | | | | | | |
| --- | --- | --- | --- | --- | --- | --- | --- | --- | --- |
|  | MVPA  Between subjects | | | MVPA  Within subjects | | | BMI | | |
| Primary endpoint^[[14]](#endnote-14)^ | Coefficient^[[15]](#endnote-15)^ | 95% CI | P-value | Coefficient^[[16]](#endnote-16)^ | 95% CI | P-value | Coefficient | 95% CI | P-value |
| Overall QoL | 2.6 | (0.3 to 4.9) | 0.031 | 1.2 | (-1.2 to 3.6) | 0.338 | -0.3 | (-0.5 to 0.01) | 0.063 |
| Physical well-being | 4.3 | (0.8 to 7.7) | 0.017 | 4.2 | (0.2 to 8.2) | 0.040 | -0.02 | (-0.4 to 0.4) | 0.923 |
| Emotional well-being | 2.7 | (-0.3 to 5.7) | 0.087 | 0.4 | (-3.0 to 3.7) | 0.836 | -0.2 | (-0.5 to 0.2) | 0.290 |
| Self-esteem | 3.4 | (-0.4 to 7.3) | 0.080 | -1.2 | (-5.5 to 3.1) | 0.590 | -0.5 | (-1.0 to -0.1) | 0.026 |
| Family connection | 1.2 | (-2.3 to 4.7) | 0.522 | -2.4 | (-5.9 to 1.1) | 0.181 | -0.4 | (-0.8 to 0.04) | 0.083 |
| Social well-being | 3.8 | (0.5 to 7.2) | 0.028 | 0.8 | (-2.8 to 4.4) | 0.663 | -0.3 | (-0.7 to 0.1) | 0.167 |
| Functioning at school | 0.1 | (-3.8 to 4.1) | 0.945 | 5.6 | (1.1 to 10.0) | 0.014 | -0.2 | (-0.6 to 0.3) | 0.430 |

**Supplement Table 4. Prospective adjusted^[[17]](#endnote-17)^ association of moderate- to-vigorous physical activity at baseline with quality of life at follow-up, additionally adjusted for body mass index^[[18]](#endnote-18)^ (SOPHYA, Switzerland, 2013 – 2019)**

| Main predictors | | | | | | |
| --- | --- | --- | --- | --- | --- | --- |
|  | MVPA | | | BMI | | |
| Primary endpoint^[[19]](#endnote-19)^ | Coefficient^[[20]](#endnote-20)^ | 95% CI | P-value | Coefficient | 95% CI | P-value |
| Overall QoL | -0.9 | (-3.5 to 1.7) | 0.488 | -0.1 | (-0.6 to 0.4) | 0.661 |
| Physical well-being | -0.7 | (-4.6 to 3.2) | 0.728 | 0.1 | (-0.6 to 0.8) | 0.834 |
| Emotional well-being | 0.1 | (-3.3 to 3.4) | 0.961 | 0.2 | (-0.4 to 0.8) | 0.528 |
| Self-esteem | 0.03 | (-4.5 to 4.6) | 0.989 | -0.5 | (-1.3 to 0.4) | 0.267 |
| Family connection | -1.0 | (-4.6 to 2.7) | 0.605 | -0.1 | (-0.8 to 0.5) | 0.693 |
| Social well-being | -0.01 | (-3.7 to 3.7) | 0.994 | -0.3 | (-1.0 to 0.4) | 0.384 |
| Functioning at school | -2.9 | (-7.5 to 1.8) | 0.226 | -0.003 | (-0.9 to 0.9) | 0.995 |

1. The exclusion criteria, which was applied to the participants at SOPHYA2 cohort, was also applied to all SOPHYA1 participants. This is why the sample size upon which the comparison was made is less than 1320 [↑](#endnote-ref-1)
2. P-value from student’s t-test [↑](#endnote-ref-2)
3. P-value from chi-squared test [↑](#endnote-ref-3)
4. Participant answered the question, but chose to abstain from declaring the range of the household income [↑](#endnote-ref-4)
5. Obtained from KINDL® questionnaire [↑](#endnote-ref-5)
6. Adjusted for age, sex, household income, language region, nationality, diagnosis with a chronic disease, season of measurement and body mass index [↑](#endnote-ref-6)
7. Sample size = 352 [↑](#endnote-ref-7)
8. Moderate- to-vigorous physical activity included in the model as moderate- to-vigorous physical activity at the respective time point [↑](#endnote-ref-8)
9. Obtained from KINDL® questionnaire [↑](#endnote-ref-9)
10. Coefficient is reflecting the change in score associated with an average 1-hour increase in moderate- to-vigorous physical activity during the accelerometry measurement week [↑](#endnote-ref-10)
11. Adjusted for age, sex, household income, language region, nationality, diagnosis with a chronic disease, season of measurement and body mass index [↑](#endnote-ref-11)
12. Sample size = 352 [↑](#endnote-ref-12)
13. Moderate- to-vigorous physical activity included in the model as participant’s mean moderate- to-vigorous physical activity over both time points (between-subject variation) and as difference from that mean or either time point (within-subject variation) [↑](#endnote-ref-13)
14. Obtained from KINDL® questionnaire [↑](#endnote-ref-14)
15. Coefficient is reflecting the change in score associated with an average 1-hour increase in between-subject moderate- to-vigorous physical activity during the accelerometry measurement week [↑](#endnote-ref-15)
16. Coefficient is reflecting the change in score associated with an average 1-hour increase in within-subject moderate- to-vigorous physical activity during the accelerometry measurement week [↑](#endnote-ref-16)
17. Adjusted for age, sex, household income, language region, nationality, diagnosed with a chronic disease, season of measurement, body mass index, and respective quality of life score at baseline [↑](#endnote-ref-17)
18. Sample size = 352 [↑](#endnote-ref-18)
19. Obtained from KINDL® questionnaire [↑](#endnote-ref-19)
20. Coefficient is reflecting the change in score associated with an average 1-hour increase in moderate- to-vigorous physical activity during the accelerometry measurement week [↑](#endnote-ref-20)
